# Pain medication management of musculoskeletal conditions at first presentation in primary care: analysis of routinely collected medical record data

**DOI:** 10.1186/1471-2474-15-418

**Published:** 2014-12-10

**Authors:** Mehluli Ndlovu, John Bedson, Peter W Jones, Kelvin P Jordan

**Affiliations:** Research Institute for Primary Care & Health Sciences, Keele University, Keele, Staffordshire ST5 5BG UK; School of Medicine, University of Nottingham, Nottingham, NG7 2UH UK; Health Services Research Unit, Keele University, Staffordshire, ST5 5NB UK

**Keywords:** Primary health care, Analgesia, Musculoskeletal, Medical records

## Abstract

**Background:**

Primary care pharmacological management of new musculoskeletal conditions is not consistent, despite guidelines which recommend prescribing basic analgesics before higher potency medications such as opioids or non-steroidal inflammatory drugs (NSAIDs).

The objective was to describe pharmacological management of new musculoskeletal conditions and determine patient characteristics associated with type of medication prescribed.

**Methods:**

The study was set within a UK general practice database, the Consultations in Primary Care Archive (CiPCA). Patients aged 15 plus who had consulted for a musculoskeletal condition in 2006 but without a musculoskeletal consultation or analgesic prescription in the previous 12 months were identified from 12 general practices. Analgesic prescriptions within two weeks of first consultation were identified. The association of socio-demographic and clinical factors with receiving any analgesic prescription, and with strength of analgesic, were evaluated.

**Results:**

3236 patients consulted for a new musculoskeletal problem. 42% received a prescribed pain medication at that time. Of these, 47% were prescribed an NSAID, 24% basic analgesics, 18% moderate strength analgesics, and 11% strong analgesics. Increasing age was associated with an analgesic prescription but reduced likelihood of a prescription of NSAIDs or strong analgesics. Those in less deprived areas were less likely than those in the most deprived areas to be prescribed analgesics (odds ratio 0.69; 95% CI 0.55, 0.86). Those without comorbidity were more likely to be prescribed NSAIDs (relative risk ratios (RRR) compared to basic analgesics 1.89; 95% CI 0.96, 3.73). Prescribing of stronger analgesics was related to prior history of analgesic medication (for example, moderate analgesics RRR 1.88; 95% CI 1.11, 3.10).

**Conclusion:**

Over half of patients were not prescribed analgesia for a new episode of a musculoskeletal condition, but those that were often received NSAIDs. Analgesic choice appears multifactorial, but associations with age, comorbidity, and prior medication history suggest partial use of guidelines.

**Electronic supplementary material:**

The online version of this article (doi:10.1186/1471-2474-15-418) contains supplementary material, which is available to authorized users.

## Background

Guidelines for the management of painful musculoskeletal conditions advocate a holistic approach incorporating education, psychological, physical, and surgical interventions administered in a step-wise fashion [1, 2]. This comprehensive strategy is, however, underpinned by the use of analgesic medications which play a central role in the management of musculoskeletal conditions [3]. In addition, musculoskeletal pain has financial consequences for both the patients and their employers in terms of sickness absence and lost productivity [4]. For example, the direct health care cost of back pain in the UK in 1998 was estimated to be around £1.6 billion with the cost of informal care and productivity losses related to back pain totalling £10 billion [4]. Currently, there is evidence that analgesic management still needs considerable improvement [5] and consequently, a better understanding of analgesic use is essential if we are to improve this element of musculoskeletal pain management.

In 2003, the World Health Organisation (WHO) offered health care professionals guidance on the use of analgesia in low back pain that is equally applicable to musculoskeletal conditions in general [1]. This advice constituted an analgesic ladder, whereby doctors were encouraged to use basic analgesics in the first instance (e.g. paracetamol), then step up to using non-steroidal anti-inflammatories if basic analgesics did not control the pain, and where appropriate as a third step, use opioid analgesics such as codeine. The WHO guidance is explicit in its use of opioids as a third line treatment because of concerns relating to the risks of addiction. This advice has been widely disseminated and is also currently reflected in the NICE guidelines for managing osteoarthritis [2].

Despite guidance advocating similar approaches for neck and low back pain, their management strategies have been shown to be different in terms of the analgesics prescribed and other therapies [6]. Whilst even with the introduction of guidelines, analgesic prescribing in patients with low back pain has not been shown to change [7]. Other influences than guidelines may affect a GP’s decision making, for example GPs may feel a more potent analgesic in the first instance is more appropriate than basic analgesics if the patient presents with severe and debilitating pain despite risk of side effects. The extent and type of analgesic prescribing in new episodes of musculoskeletal pain is unclear, as is how it varies by site of problem (for example, knee, hip, back), or whether there are characteristics of the patient which are associated with the decision to prescribe analgesia and the type of analgesia prescribed.

The primary objectives of this study were therefore, to describe the analgesics that primary care clinicians prescribe when a patient consults with a new episode of a musculoskeletal condition, and to determine socio-demographic and clinical factors associated with being prescribed medication and type of medication.

## Methods

### Population

This was a retrospective study based in the Consultations in Primary Care Archive (CiPCA). CiPCA is a high quality primary care database, giving comparable musculoskeletal consultation prevalence to national primary care databases [8, 9]. Approval to download and store anonymised medical record information for research was granted by the North Staffordshire Research Ethics Committee. All general practices participating in CiPCA inform their registered patients that their anonymised records will be used in this way. Patients are offered the opportunity to withdraw their records from inclusion in CiPCA. In the UK, the majority of the population are registered with a general practice, and this is normally the first point of access to the National Health Service. Data was used from 12 general practices which contributed data for the period 2004–2006.

Patients aged 15 and over were included if they consulted for a musculoskeletal condition in 2006, with no musculoskeletal consultation and no prescribed analgesic medication for the 12 months preceding their 2006 consultation. Musculoskeletal conditions were defined using Read Codes based on a previously derived set of codes [10]. Read codes are a commonly used system for recording morbidity in UK primary care. Musculoskeletal consultations were identified as those recorded with any Read code within Chapter N “Musculoskeletal and connective tissues diseases” or with Read codes considered by consensus of 2 GPs to be musculoskeletal in nature within Chapters R “Symptoms, Signs and Ill-defined conditions”, and 1 "History/Symptoms". Four GPs then allocated all such Read codes to individual body regions (e.g. back, knee) or unspecified if no region could be allocated. “Unspecified” problems tended to be codes where either no region was described in the associated Read Term (e.g. the term simply specified “arthralgia”) or the problem covered more than one region (e.g. “generalised osteoarthritis”). Injuries were excluded in order to focus on musculoskeletal conditions which have no obvious aetiology and have the potential for becoming chronic complaints. In the majority of cases injuries tend to be self-limiting and of relatively short duration. The inclusion criterion is based on the assumption that any patient who does not consult for a musculoskeletal condition and does not receive prescribed analgesic medication for 12 months does not have a chronic or persistent musculoskeletal problem that is currently considered by the patient as troublesome [11].

### Pain medication

**Figure 1:**
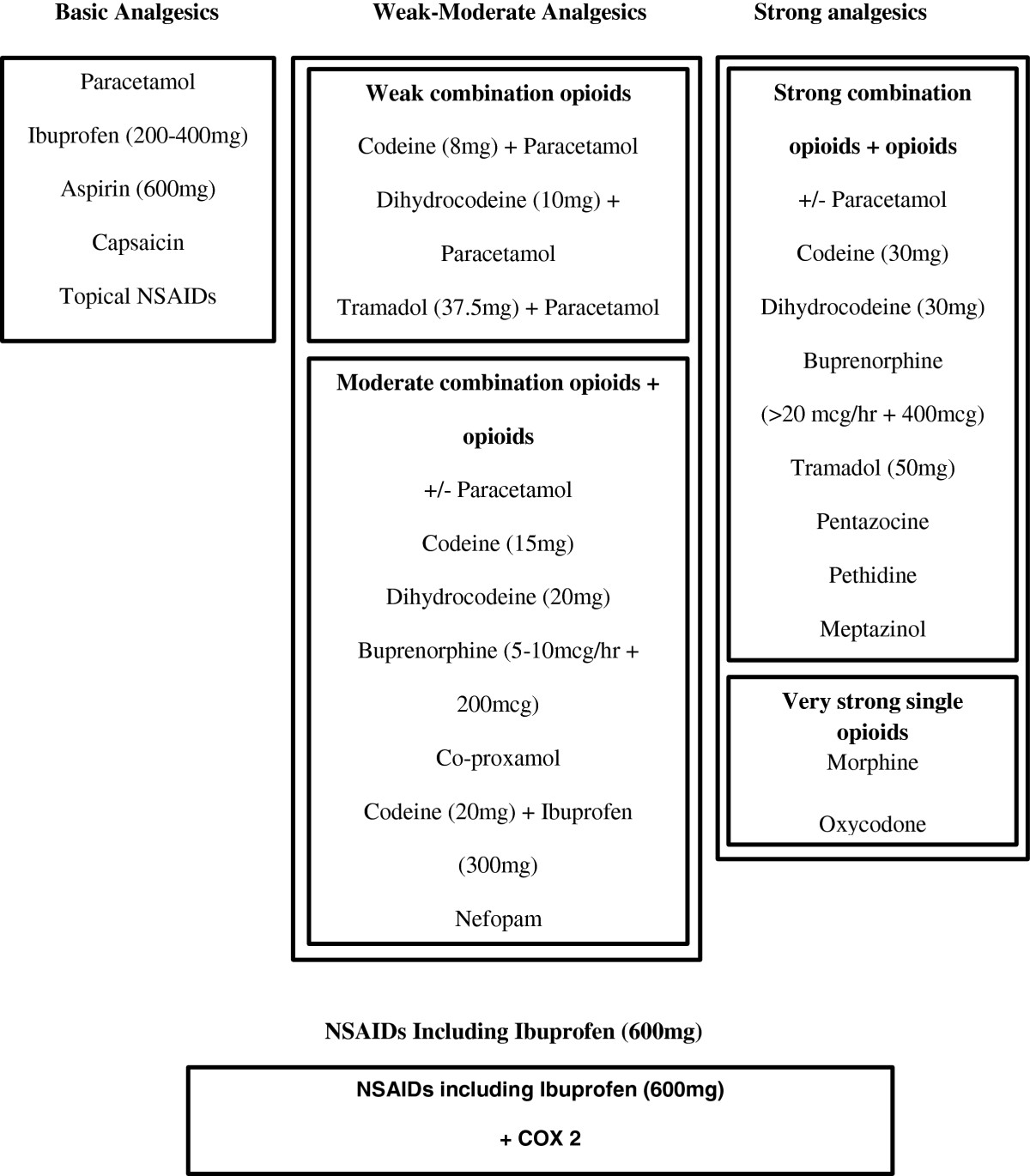
**Revised hierarchical analgesic categorisation model for prescribing analgesics and NSAIDs in primary care.** Modified version of that published in Bedson J, Belcher J, Martino OI et al. The effectiveness of national guidance in changing analgesic prescribing in primary care from 2002 to 2009: an observational database study, European Journal of Pain, 2013;17:434–443, with permission of John Wiley and Sons.

In the United Kingdom (UK) there are over 300 prescribable analgesic formulations available to general practitioners (GPs) [12]. Bedson and colleagues, using GPs in a consensus exercise, derived a hierarchical analgesic categorisation where all analgesic formulations were categorised into six groups according to equipotency levels when treating varying levels of perceived pain as shown in Figure 1[13]. Group 1 comprises basic analgesics such as paracetamol. Groups 2–4 are made up of increasingly potent opioids either alone or in combination with paracetamol. Group 2 includes weak opioids (for example, codeine 8 mg). Group 3 includes moderate opioids (for example, codeine 15 mg). Group 4 includes strong opioids (for example, codeine 30 mg). Group 5 contains very strong opioids (morphine and oxycodone). Group 6 comprises non-steroidal anti-inflammatory drugs (NSAIDs) which the consensus exercise did not rank in the potency ladder but were considered as an adjunct to analgesic prescribing.

For this study, due to the low prevalence of prescription of very strong opioids [13] and to aid interpretation, the groupings were simplified into four main groups by combining weak and moderate combination opioids (weak-moderate analgesics), and strong combination opioids with very strong single opioids (strong analgesics)*.* Pain medication was deemed as being related to a musculoskeletal consultation if it was prescribed on the same day as the consultation, or within 14 days of that consultation. This was to allow for patients to take up a ‘delayed’ prescription for analgesics that the GP offered should the condition not improve.

### Socio-demographic and clinical factors

Factors evaluated for their association with pain medication prescription on first consultation included the patient’s age, gender, co-morbidity, region of pain, previous consultation for musculoskeletal conditions, analgesic medication history, neighbourhood deprivation, registered practice, and staff consulted. The age of the patient was calculated as of 1st July 2006 and grouped into the following categories: 15–29, 30–44, 45–59, 60–74, and 75+. Neighbourhood deprivation was based on the Index of Multiple Deprivation 2007. This is linked to the postcodes of patient addresses [14]. The deprivation ranks range from 1 to 32482, with 1 being the most deprived neighbourhood and 32482 least deprived in England. This variable was categorised into 3 levels with patients in the lower third based on deprivation rank being the most deprived, the middle third moderately deprived and the top third least deprived. The staff member consulted was categorised into GPs and all other medical staff (such as practice nurses and nurse practitioners).

The area of pain was categorised as back, knee, hip, neck, foot and ankle, arm (hand, wrist, arm, elbow and upper limb), shoulder, and other or unspecified. The specified areas are the most common locations of musculoskeletal pain [15]. Identification of pain region used a previously derived classification [10]. Previous consultation for a musculoskeletal problem was defined as having a recorded musculoskeletal consultation in the period 12 to 24 months before the baseline musculoskeletal consultation. Similarly, previous pain medication was defined as receiving any prescribed pain medication 12 to 24 months before the baseline musculoskeletal consultation. These variables give a brief medical history of the patient which clinicians may consider in deciding whether to give medication or not and which medication to prescribe [16].

Co-morbidity was defined as the presence of one or more specified disorders or diseases in the period 0–24 months before the baseline musculoskeletal consultation. The specific comorbidities were diabetes, chronic obstructive pulmonary disease (COPD), depression, cardiovascular disease, chronic kidney disease, gastro-intestinal, and neoplasm. These are long term comorbidities which clinicians take into consideration when deciding which type of analgesia to prescribe [16].

### Statistical analysis

Multilevel logistic regression was used to evaluate the associations of being prescribed any pain medication on first consultation with patient and practice characteristics. Levels within the multilevel model were patient (level 1) within practice (level 2). Results are presented as odds ratios (OR) with 95% confidence intervals. Both adjusted and unadjusted odds ratios for the patient characteristics are reported.

A second analysis was then performed on only those receiving a pain medication. A multilevel multinomial logistic regression model with the analgesic group as the dependent variable was used to assess associations of patient and practice characteristics with type of analgesic received. The reference category was group 1 (basic analgesics). Results are reported as relative risk ratios (RRR) with 95% confidence intervals. All models were evaluated using a 5% statistical significance level with no adjustment for multiple testing using the statistical package Stata.

## Results

### New consulters for musculoskeletal problems

In 2006, there were 83,875 patients aged 15 and over registered at the 12 practices. 3236 (386 per 10,000) patients were identified as having a new consultation for a musculoskeletal problem in 2006. The mean age of these 3236 patients was 43.1 years (SD 15.8), and 1916 (59%) were male (Table 1).

**Table 1:** **Socio-demographic and clinical characteristics of patients with new consulting episode of musculoskeletal pain in 2006**

			Analgesic received	
	N	Column %	n	Row %
Total	3236	-	1344	42
Age (years)				
15-29	710	22	224	32
30-44	1081	33	448	41
45-59	963	30	428	44
60-74	374	12	181	48
75+	108	3	63	58
Gender				
Females	1320	41	551	42
Males	1916	59	793	41
Previous musculoskeletal consultation^a^				
Yes	413	13	163	39
No	2823	87	1181	42
Previous analgesic prescription^b^				
Yes	512	16	244	48
No	2724	84	1100	40
Region of Pain				
Back	838	26	465	55
Knee	341	11	144	42
Hip	203	6	68	33
Foot and Ankle	223	7	94	42
Arm	314	10	105	33
Shoulder	245	8	129	53
Neck	218	7	93	43
Other/unspecified	854	26	246	29
Co-morbidity^c^				
Yes	119	4	63	53
No	3117	96	1281	41
Deprivation				
Most	1430	44	624	44
Moderate	1263	39	508	40
Least	543	17	212	39
Staff category				
GPs	2616	81	1117	43
Other	620	19	227	37
Practice				
1	254	8	79	31
2	224	7	78	35
3	219	7	77	35
4	253	8	76	30
5	161	5	59	37
6	283	9	115	41
7	420	13	241	57
8	324	10	143	44
9	288	9	130	45
10	327	10	166	51
11	143	4	57	40
12	340	11	123	36

N = Total number of patients for each category, n = number of patients prescribed analgesic per category, Col. % = N/3236, Row % = n/N for each row.

^a^musculoskeletal consultation 12–24 months before their 2006 consultation.

^b^prescribed pain medication 12–24 months before their 2006 consultation.

^c^diabetes, chronic obstructive pulmonary disease (COPD), depression, cardiovascular disease, chronic kidney disease, gastro-intestinal, or neoplasm.

The back was the most common site of the musculoskeletal problem (26%), followed by the knee (11%). Least commonly affected was the hip (6%), with other or unspecified regions accounting for 26% (Table 1). 13% had a recent history of consulting for a musculoskeletal problem, albeit not within the previous 12 months, and 16% had received pain medication 12–24 months before their 2006 consultation.

### Pain medication prescriptions

1344 (42%) patients received prescribed pain medication within 14 days of their new musculoskeletal consultation (Table 1). Of those who received prescribed pain medication, 24% received basic analgesics, 18% weak or moderate analgesics, 11% strong analgesics and 47% NSAIDs (Table 2). Socio-demographic and clinical characteristics of those prescribed the different types of pain medication are detailed in Table 2.

**Table 2:** **Socio-demographic and clinical characteristics of patients prescribed each group of analgesic**

	Pain medication prescribed							
	Basic analgesic		Weak/moderate analgesic		NSAIDs		Strong analgesic	
	n	%	n	%	n	%	n	%
Total	321	24	239	18	637	47	147	11
	*n*	*%*	*n*	*%*	*n*	*%*	*n*	*%*
Age (years)								
15-29	85	26	28	12	96	15	15	10
30-44	73	23	66	28	250	39	59	40
45-59	79	24	77	32	221	35	51	35
60-74	53	17	44	18	65	10	19	13
75+	31	10	24	10	5	1	3	2
Gender								
Females	137	43	118	49	230	36	66	45
Males	184	57	121	51	407	64	81	55
Previous musculoskeletal consultation^a^								
Yes	33	10	28	12	83	13	19	13
No	288	90	211	88	554	87	128	87
Previous analgesic prescription^b^								
Yes	40	12	49	21	127	20	28	19
No	281	88	190	79	510	80	119	81
Region of Pain								
Back	64	20	114	48	203	32	84	57
Knee	44	14	11	5	84	13	5	3
Hip	10	3	13	5	38	6	7	5
Foot and Ankle	26	8	10	4	54	9	4	3
Arm	41	13	9	4	54	8	1	1
Shoulder	34	10	14	6	71	11	10	7
Neck	17	5	15	6	44	7	17	12
Other/unspecified	85	26	54	22	89	14	19	13
Co-morbidity^c^								
Yes	20	6	13	5	22	3	8	5
No	301	94	226	95	615	97	139	95
Deprivation								
Most	146	46	123	52	277	43	78	53
Moderate	120	37	82	34	254	40	52	35
Least	55	17	34	14	106	17	17	12
Staff category								
GPs	273	85	210	88	523	82	111	76
Other	48	15	29	12	114	18	36	24

Total % = n/1344, *%* = *n*/n.

^a^musculoskeletal consultation 12–24 months before their 2006 consultation.

^b^prescribed pain medication 12–24 months before their 2006 consultation.

^c^diabetes, chronic obstructive pulmonary disease (COPD), depression, cardiovascular disease, chronic kidney disease, gastro-intestinal, or neoplasm.

### Factors associated with prescription of analgesics

The associations of prescribing any pain medication with socio-demographic and clinical factors are shown in Table 3. There was significant practice variation in the decision to prescribe (range across practices of 30% to 57% of patients receiving analgesics) with variation between practices accounting for 9% of unexplained variation in patients being prescribed analgesics in the multivariable model.

**Table 3:** **Associations with prescription of any analgesic at new consultation for musculoskeletal pain**

Model	OR [95% CI]		
Fixed effects	Unadjusted	Adjusted	Adjusted ***p***-value
Age Group			
30-44	1.00	1.00	-
15-29	0.65 [0.53, 0.80]	0.69 [0.56, 0.85]	<0.001
45-59	1.12 [0.94, 1.34]	1.23 [1.02, 1.49]	0.025
60-74	1.28 [1.01, 1.64]	1.51 [1.17, 1.95]	0.002
75+	1.85 [1.23, 2.78]	2.28 [1.49, 3.49]	<0.001
Gender			
Male	1.00	1.00	-
Female	1.00 [0.87, 1.15]	1.02 [0.88, 1.19]	0.780
Previous musculoskeletal consultation^a^			
No	1.00	1.00	-
Yes	0.89 [0.72, 1.09]	0.83 [0.66, 1.05]	0.119
Previous analgesic prescription^b^			
No	1.00	1.00	-
Yes	1.24 [1.03, 1.49]	1.24 [1.01, 1.54]	0.045
Pain Region			
Back	1.00	1.00	-
Knee	0.59 [0.46, 0.77]	0.56 [0.43, 0.72]	<0.001
Hip	0.38 [0.27, 0.52]	0.35 [0.25, 0.49]	<0.001
Foot and Ankle	0.55 [0.40, 0.74]	0.52 [0.38, 0.71]	<0.001
Arm	0.40 [0.30, 0.54]	0.39 [0.29, 0.53]	<0.001
Shoulder	0.90 [0.67, 1.20]	0.81 [0.60, 1.08]	0.155
Neck	0.58 [0.43, 0.79]	0.57 [0.42, 0.78]	<0.001
Other/unspecified	0.32 [0.26, 0.39]	0.29 [0.24, 0.37]	<0.001
Comorbidity^c^			
Yes	1.00	1.00	-
No	0.64 [0.44, 0.92]	0.77 [0.52, 1.13]	0.217
Deprivation			
Most	1.00	1.00	-
Medium	0.85 [0.73, 1.01]	0.80 [0.67, 0.95]	0.012
Least	0.77 [0.63, 0.95]	0.69 [0.55, 0.86]	0.001
Staff category			
Other	1.00	1.00	-
GP	0.85 [0.70, 1.02]	0.85 [0.69, 1.04]	0.108
Random effect		VARIANCE	
Practice	0.08 [0.03, 0.21]	0.09 [0.03, 0.23]	<0.001

^a^musculoskeletal consultation 12–24 months before their 2006 consultation.

^b^prescribed pain medication 12–24 months before their 2006 consultation.

^c^diabetes, chronic obstructive pulmonary disease (COPD), depression, cardiovascular disease, chronic kidney disease, gastro-intestinal, or neoplasm.

Compared to the 30–44 year old age group, the odds of being prescribed pain medication on first consultation were significantly less in those aged 15 to 29 (adjusted OR 0.69; 95% CI 0.56, 0.85) but higher in those aged 45 to 59 (OR 1.23; 95% CI 1.02, 1.49), 60 to 74 (OR 1.51; 95% CI 1.17, 1.95), and those aged over 75 (OR 2.28; 95% CI 1.49, 3.49). Those in the least deprived areas were least likely to receive a pain medication prescription (OR 0.69; 95% CI 0.55, 0.86). Pain medication was most likely to be prescribed for those with pain in the back; however, no difference was apparent between those with shoulder and back problems. Those who had received prescribed analgesics in the past were more likely to be prescribed pain medication at this new consultation (OR 1.24; 95% CI 1.01, 1.54).

There were no significant relationships with comorbidity, gender or whether the patient saw a GP or other medical staff.

### Factors associated with type of analgesics

In the 1344 patients prescribed analgesics, there was wide variation between practices in type of analgesic prescribed. Variation between practices accounted for 27% of all remaining variation in type of medication prescribed in the multivariable model.

Table 4 shows the associations of socio-demographic and clinical factors with type of analgesics in those prescribed analgesics. Compared to those aged 30–44, patients aged 15–29 were more likely to receive basic analgesics than weak-moderate analgesics, strong analgesics or NSAIDs. In the case of NSAIDs for example, for patients aged 15–29 the adjusted RRR was 0.30 (95% CI 0.20, 0.46) compared to the 30–44 age group. A similar decreased chance of NSAID prescription was also evident in those aged over 60 (for example, aged 75 and above, RRR 0.05; 95% CI 0.02, 0.13). Females were more likely than males to be prescribed weak-moderate analgesics compared to basic analgesics (RRR 1.45; 95% CI 1.02, 2.09). A previous history of analgesic prescription was associated with the prescribing of stronger medication compared to basic analgesics (for example, weak-moderate analgesic, RRR 1.88; 95% CI 1.11, 3.10). Strong analgesics were less likely to be prescribed than basic analgesics to those living in the least deprived areas (RRR 0.45; 95% CI 0.23, 0.88). There was a marginally non-significant increased likelihood of being prescribed NSAIDs if the patient did not have comorbidity (RRR 1.89; 95% CI 0.96, 3.73).

**Table 4:** **Associations with type of analgesia prescribed at new consultation for musculoskeletal pain in those prescribed an analgesic**

Model		RRR [95% CI]					
Fixed effects		Weak-moderate analgesics		Strong analgesics		NSAIDs	
	***n***	Unadjusted	Adjusted	Unadjusted	Adjusted	Unadjusted	Adjusted
Age Group							
30-44	224	1.00	1.00	1.00	1.00	1.00	1.00
15-29	448	0.33 [0.19, 0.57]	**0.32 [0.18, 0.57]**	0.20 [0.10, 0.38]	**0.20 [0.10, 0.40]**	0.30 [0.20, 0.45]	**0.30 [0.20, 0.46]**
45-59	428	1.07 [0.67, 1.17]	1.32 [0.81, 2.15]	0.79 [0.48, 1.30]	1.04 [0.62, 1.78]	0.81 [0.56, 1.18]	0.83 [0.54, 1.22]
60-74	181	0.87 [0.51, 1.46]	1.19 [0.67, 2.13]	0.42 [0.22, 0.78]	0.71 [0.35, 1.42]	0.34 [0.21, 0.53]	**0.35 [0.22, 0.57]**
75+	63	0.76 [0.40, 1.47]	1.01 [0.50, 2.05]	0.11 [0.03, 0.37]	**0.18 [0.05, 0.64]**	0.04 [0.02, 0.11]	**0.05 [0.02, 0.13]**
Gender							
Male	793	1.00	1.00	1.00	1.00	1.00	1.00
Female	551	1.32 [0.94, 1.86]	**1.45 [1.02, 2.09]**	1.10 [0.74, 1.62]	1.31 [0.85, 2.01]	0.77 [0.58, 1.01]	0.95 [0.70, 1.28]
Previous musculoskeletal consultation^a^							
No	1181	1.00	1.00	1.00	1.00	1.00	1.00
Yes	163	1.10 [0.64, 1.90]	0.86 [0.47, 1.58]	1.23 [0.67, 2.27]	0.98 [0.49, 1.95]	1.24 [0.80, 1.93]	0.93 [0.57, 1.54]
Previous analgesic prescription^b^							
No	1100	1.00	1.00	1.00	1.00	1.00	1.00
Yes	244	1.64 [1.03, 2.61]	**1.88 [1.11, 3.10]**	1.49 [0.88, 2.56]	1.72 [0.94, 3.16]	1.58 [1.07, 2.35]	**1.74 [1.11, 2.71]**
Pain Region							
Back	465	1.00	1.00	1.00	1.00	1.00	1.00
Knee	144	0.13 [0.06, 0.26]	**0.11 [0.05, 0.23]**	0.08 [0.03, 0.21]	**0.08 [0.03, 0.23]**	0.54 [0.34, 0.87]	0.65 [0.40, 1.08]
Hip	68	0.72 [0.29, 1.77]	0.61 [0.24, 1.55]	0.53 [0.19, 1.48]	0.62 [0.21, 1.81]	1.18 [0.55, 2.56]	1.59 [0.71, 3.55]
Foot and Ankle	94	0.21 [0.09, 0.47]	**0.17 [0.07, 0.39]**	0.11 [0.04, 0.35]	**0.10 [0.03, 0.32]**	0.63 [0.37, 1.12]	0.61 [0.34, 1.10]
Arm	105	0.12 [0.05, 0.26]	**0.10 [0.05, 0.23]**	0.02 [0.00, 0.14]	**0.02 [0.00, 0.13]**	0.40 [0.24, 0.67]	**0.40 [0.24, 0.69]**
Shoulder	129	0.22 [0.11, 0.45]	**0.18 [0.09, 0.37]**	0.21 [0.10, 0.47]	**0.18 [0.08, 0.40]**	0.62 [0.38, 1.05]	**0.56 [0.33, 0.96]**
Neck	93	0.49 [0.23, 1.06]	0.50 [0.23, 1.12]	0.76 [0.35, 1.61]	0.83 [0.37, 1.84]	0.81 [0.43, 1.54]	0.82 [0.42, 1.62]
Other/unspecified	246	0.36 [0.22, 0.57]	**0.32 [0.20, 0.53]**	0.17 [0.10, 0.32]	**0.18 [0.10, 0.34]**	0.34 [0.22, 0.51]	**0.38 [0.25, 0.59]**
Comorbidity^c^							
Yes	63	1.00	1.00	1.00	1.00	1.00	1.00
No	1281	1.18 [0.57, 2.44]	1.49 [0.68, 3.26]	1.18 [0.50, 2.76]	1.40 [0.56, 3.53]	1.89 [1.30, 3. 57]	1.89 [0.96, 3.73]
Deprivation							
Most	624	1.00	1.00	1.00	1.00	1.00	1.00
Medium	508	0.85 [0.58, 1.26]	0.75 [0.49, 1.13]	0.85 [0.58, 1.33]	0.76 [0.47, 1.22]	1.17 [0.86, 1.61]	1.19 [0.85, 1.67]
Least	212	0.74 [0.44, 1.23]	0.58 [0.33, 1.01]	0.58 [0.31, 1.09]	**0.45 [0.23, 0.88]**	1.02 [0.68, 1.54]	1.00 [0.64, 1.56]
Staff category							
Other	227	1.00	1.00	1.00	1.00	1.00	1.00
GP	1117	0.79 [0.47, 1.04]	0.81 [0.47, 1.39]	1.86 [1.12, 3.09]	**1.74 [1.01, 3.02]**	1.25 [0.84, 1.86]	1.17 [0.77, 1.79]
Random effect	Adjusted VARIANCE [standard error]						
Practice		0.27 [0.13]					

^a^musculoskeletal consultation 12–24 months before their 2006 consultation.

^b^prescribed pain medication 12–24 months before their 2006 consultation.

^c^diabetes, chronic obstructive pulmonary disease (COPD), depression, cardiovascular disease, chronic kidney disease, gastro-intestinal, or neoplasm.

Bold indicates *p* < 0.05 in adjusted analysis. Reference is prescription of basic analgesic.

Those with back pain were more likely to be prescribed weak-moderate analgesics, strong analgesics and NSAIDs than basic analgesics compared to those presenting with musculoskeletal problems in other regions. Those who were seen by GPs were more likely to be prescribed strong analgesics than basic analgesics (RRR 1.74; 95% CI 1.01, 3.02).

## Discussion

This study has shown that more than half the patients consulting for a new episode of a musculoskeletal condition were not prescribed pain medication. Of those who were, most commonly NSAIDs were prescribed, with basic analgesics being the second most common analgesic. There appears to be restricted early use of stronger medication with strong opioids being prescribed for only one in ten new musculoskeletal patients prescribed pain medication. Patient age, deprivation, body region of pain, comorbidity and previous analgesic prescriptions were associated with prescribing behaviour and there was extensive practice variation.

Our study found less than half of new consulters for musculoskeletal pain received an analgesic. A previous study of those aged over 50 consulting in primary care for musculoskeletal pain, and who had not consulted in the previous 30 days, also reported that less than half were prescribed analgesics [17] and this is also similar to findings from studies focussed on neck and back pain [6, 7] and on osteoarthritis [18]. It is feasible that GPs may be following guidelines that recommend the early use of exercise and other physical therapies with or without analgesia and further research is needed to determine if this is happening [1, 19].

The WHO advice relating to managing low back pain, one of the commonest forms of musculoskeletal pain treated in primary care [1], suggested that clinicians should administer simple analgesics like paracetamol for pain relief prior to considering using oral NSAIDs, and then opioids if pain is not controlled by the previous analgesic group. Basic analgesics are available over the counter without prescription, and patients first seen in primary care may already be using these medications. Prescribing of NSAIDs may reflect clinicians feeling that the severity of their patients’ complaints warrants an analgesic stronger than that available without prescription. If a basic analgesic has already been used, the next step in the WHO analgesic ladder is to use NSAIDs, followed by weak opioids and subsequently stronger ones. In our study the most potent opioid medications were indeed prescribed less than basic analgesics on first consultation. This may also reflect other elements of the clinician’s decision making process, which takes into account the adverse side effects that patients are more likely to experience when prescribed higher potency drugs. Starting at a lower potency minimises this risk [16, 20, 21]. Rather than a patient’s previous consultation history for musculoskeletal conditions, it appeared that the clinician’s knowledge of previous analgesic medication affected their prescribing. A patient with a past history of prescribed pain medication was more likely to receive any analgesic and, particularly, stronger analgesic, as has been shown previously [17]. Clinicians will generally ask the patient about their medication use prior to consultation [20]. The more potent analgesic may subsequently be used if the patient’s experience of pain relief with the previous basic analgesic was unsatisfactory.

Clinicians appear to consider patient age when choosing to prescribe a pain medication or not, and what level of analgesic potency to prescribe. In our study, those aged over 75 were twice as likely to receive pain medication as younger age groups, but were less likely to be prescribed NSAIDs. This finding is in keeping with current advice on NSAID use in older patients who might be considered more likely to experience adverse effects such as renal toxicity [16, 22] and gastrointestinal haemorrhage [23, 24] with NSAIDs. Stronger opioids were less likely to be used in those aged over 75, a finding that has also been described previously [25–28]. This would make clinical sense since using these more potent opioid type drugs in the elderly has been associated with increased rates of falls and bone fractures [21]. Comorbidity was linked to a lower likelihood of being prescribed NSAIDs which also reflects the possibility clinicians are avoiding these drugs in patients more vulnerable to side effects [29]. However, this finding was non-significant and the prevalence of our selected comorbidities in this group with new musculoskeletal problems was low. Further research is needed on the extent to which comorbidity influences analgesic prescribing.

Younger adults (15–29) were more likely to be prescribed basic analgesics, perhaps reflecting the less severe nature of pain in younger people with musculoskeletal problems [30]. Female patients were more likely to be prescribed weak or moderate opioids over basic analgesics. They also had a non-significant higher likelihood of receiving stronger analgesics. Females are often perceived as experiencing more pain than males and females have been shown to be better than males at communicating their pain [3, 20, 31, 32], which may influence the decision to prescribe more potent medication than basic analgesics.

Patients from the most deprived areas were more likely to be prescribed pain medication than patients from medium and least deprived areas and be prescribed stronger analgesics. Social characteristics such as neighbourhood level of deprivation have an additional effect on pain [33, 34]. The level of pain is associated with emotional distress, low social support and low social participation that may be more common in deprived areas [20, 33]. Further, patients in more deprived areas may rely on prescribed medication even for pain that can be eased with over the counter medications as prescriptions are free for low income and older patients in the UK [35]. Patients from least deprived areas may prefer to purchase over the counter medications as they pay for their prescriptions, a finding shown previously for over the counter use of aspirin in cardiovascular diseases [36].

Patients presenting with back problems were most likely to receive pain medication and to receive stronger analgesics. Back pain limits the functional reach of limbs and the ability to rotate the trunk repetitively which is essential for mobility, and results in restrictions on individuals’ social and physical activities and a substantial impact on their life style [32]. Clinicians may be more likely to perceive back pain as limiting in the day to day activities that a person has to perform hence consider there is a greater need to prescribe stronger pain medication for back pain. Additionally, clinicians’ perceptions of the handicap that pain causes may vary across different body regions. For example back, knee and hip pain may be perceived as being debilitating in terms of mobility, whilst shoulder, wrist and hand pain limit daily activities such as washing, cooking, and cleaning [15, 37, 38]. This may be reflected in some body locations being associated with prescription of more potent analgesics than others.

The findings of this study are limited by the fact that it was based in a regional dataset and therefore might not be reflective of analgesic prescribing in other areas of the United Kingdom. However, the data used in the study is drawn from a data set, CiPCA, which gave comparable consultation figures for musculoskeletal conditions as the larger national databases [8]. We did not evaluate the prescribing of pain medication by specific diagnoses. However, patients often present to primary care with a regional musculoskeletal symptom, such as back or knee pain, which is not initially labelled with a diagnosis. Region specific management is common in primary care, for example for back pain [1], and GPs may prefer to work with a regional pain label than a complex diagnostic label. Patients were selected over a 12 month consultation period and the inclusion criteria may include patients with musculoskeletal episodes with a periodicity more than 12 months and those who have been taking over the counter medications or receiving other care. Their previous consultation and medication prescription history may not be a true reflection of the starting point of the pharmacological management of a musculoskeletal condition. However the inclusion criteria ensures that it is reasonable to consider the patients as having no chronic pain prior to consulting as chronic pain is defined as pain lasting more than three months [5, 11]. Clinicians consider multiple factors in deciding the medication and appropriate dose [16, 20, 39, 40] and there may be other unmeasured variables such as other contraindications which may influence prescribing. Pain severity, weight, alcohol misuse and ethnicity might also impact on prescription of pain medication [41] and these are not evaluated in this analysis.

## Conclusion

This study highlights that age, deprivation, body region of pain, comorbidity and previous analgesic prescriptions are associated with analgesic prescribing in primary care when treating patients with a new consulting episode of a musculoskeletal condition. There appears to be a clinically sensible use of these painkillers, in as much as analgesics such as NSAIDs and opioids are less likely to be used in groups that may be more vulnerable to potential side effects. Prescribing also varies depending upon the region of pain being treated. The prescribing characteristics may follow the WHO analgesic ladder of prescribing since there is a restricted use of stronger analgesics at first consultation. However, further research into the pharmacological management of musculoskeletal pain is required to determine if these patterns of prescribing represent the optimum management pathways when managing musculoskeletal conditions.

## Authors’ original submitted files for images

Below are the links to the authors’ original submitted files for images. Authors’ original file for figure 1

## Abbreviations

CI: Confidence interval
CiPCA: Consultations in Primary Care Archive
COPD: Chronic obstructive pulmonary disease
GP: General practitioner
mg: Milligram
NICE: National Institute for Health and Care Excellence
NSAID: Non-steroidal anti-inflammatory drug
OR: Odds ratio
RRR: Relative risk ratio
SD: Standard deviation
UK: United Kingdom
WHO: World Health Organization.
