# Supplementary material for: Pain medication management of musculoskeletal conditions at first presentation in primary care: analysis of routinely collected medical record data
Source: BMC Musculoskelet Disord. 2014 Dec 10;15:418. doi: 10.1186/1471-2474-15-418 (PMC4295256; doi:10.1186/1471-2474-15-418)
Supplement: Supplementary file 1 — Authors’ original file for figure 1 [file 12891_2014_2351_MOESM1_ESM.pdf]

Basic Analgesics

Paracetamol

Ibuprofen (200-400mg)

Aspirin (600mg)

Capsaicin

Topical NSAIDs

Weak-Moderate Analgesics

Weak combination opioids

Codeine (8mg) + Paracetamol

Dihydrocodeine (10mg) + Paracetamol

Tramadol (37.5mg) + Paracetamol

Moderate combination opioids + opioids

+/- Paracetamol

Codeine (15mg)

Dihydrocodeine (20mg)

Buprenorphine (5-10mcg/hr + 200mcg)

Co-proxamol

Codeine (20mg) + Ibuprofen (300mg)

Nefopam

Strong analgesics

Strong combination opioids + opioids

+/- Paracetamol

Codeine (30mg)

Dihydrocodeine (30mg)

Buprenorphine (>20 mcg/hr + 400mcg)

Tramadol (50mg)

Pentazocine

Pethidine

Meptazinol

Very strong single opioids

Morphine

Oxycodone

NSAIDs Including Ibuprofen (600mg)

NSAIDs including Ibuprofen (600mg)

+ COX 2
